# BFP, bifacial weakness with paresthesias secondary to trauma: a case report

**DOI:** 10.3389/fncel.2026.1916633

**Published:** 2026-09-03

**Authors:** Jingjing Chen, Xuxia Tang, Shuo Dai, Xiao He

**Affiliations:** Department of Otolaryngology, The First Affiliated Hospital of Zhejiang Chinese Medical University (Zhejiang Provincial Hospital of Chinese Medicine), Hangzhou, China

**Keywords:** BFP, bifacial weakness with paresthesias, bilateral facial paralysis, Guillain-Barré syndrome, trauma

## Abstract

**Background:**

BFP, bifacial weakness with paresthesias is a rare variant of Guillain-Barré syndrome (GBS). Here, we detail the diagnosis and treatment process of a patient with BFP secondary to head trauma to highlight its importance.

**Case:**

A 47-year-old man presented with bilateral peripheral facial paralysis, hearing loss, and sensory impairment in both lower limbs following a car accident. Temporal bone fracture was first considered as the cause of facial palsy. We gave glucocorticoid therapy to the patient, but it was ineffective, facial nerve decompression surgery was planned as a consideration. However, diminished tendon reflexes was noted on the 12th day post-trauma, then cerebrospinal fluid (CSF) testing and anti-ganglioside antibodies were performed. Based on the medical history and Laboratory testing, the patient was diagnosed with BFP, a rare variant of GBS. Plasma exchange therapy was performed on the patient. At six-month follow-up, except for hearing loss, the other symptoms had almost returned to normal.

**Conclusion:**

In this article, we report a case of BFP secondary to head trauma. We emphasize that in patients with post-traumatic bilateral facial nerve paralysis, a comprehensive neurological examination is essential for accurate diagnosis and to avoid unnecessary medications or surgery.

## 1 Introduction

Guillain-Barré syndrome (GBS) is an acute immune-mediated polyneuropathy characterized by rapidly progressive symmetric limb weakness and diminished or absent tendon reflexes, often accompanied with sensory disturbances. Its pathogenesis is closely related to damage to the peripheral nerve myelin or axons caused by immune-mediated mechanisms. In addition to the classic form, it includes several variants characterized by local involvement. BFP, bifacial weakness with paresthesias is a rare localized variant of GBS, characterized by acute-onset symmetric or mildly asymmetric bilateral facial nerve paralysis as the predominant manifestation, accompanied by distal limb paresthesias, with normal or only mildly reduced tendon reflexes. The challenge of diagnosing BFP is that mild findings—such as distal limb paresthesias suggestive of systemic polyneuropathy—may easily be overlooked in patients with prominent facial palsy. When BFP is secondary to head trauma, the diagnostic process becomes even more challenging, as temporal bone fracture is a common cause of facial palsy. In such cases, ENT and emergency departments often serve as the initial points of contact, however, subtle sensory abnormalities may be missed, leading to an initial diagnosis that leans toward traumatic facial nerve injury.

We report a case of post-traumatic BFP to underscore that, when bilateral facial paralysis is encountered after head trauma, clinicians must remain highly vigilant, necessitating detailed history taking and comprehensive physical examinations.

## 2 Case report

**FIGURE 1:**
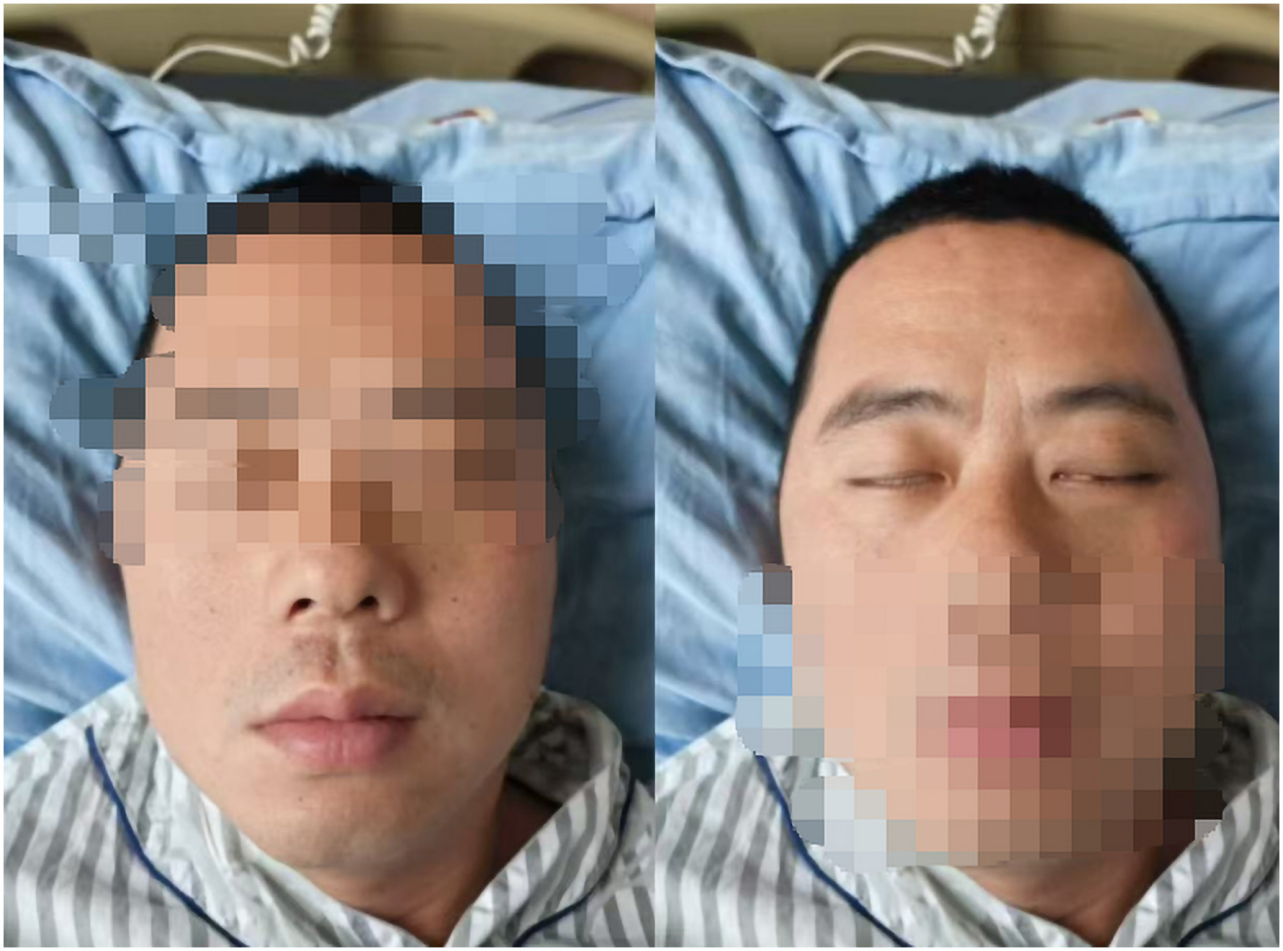
Seven days after trauma, the patient presented with incomplete eye closure, flattening of the nasolabial fold.

**FIGURE 2:**
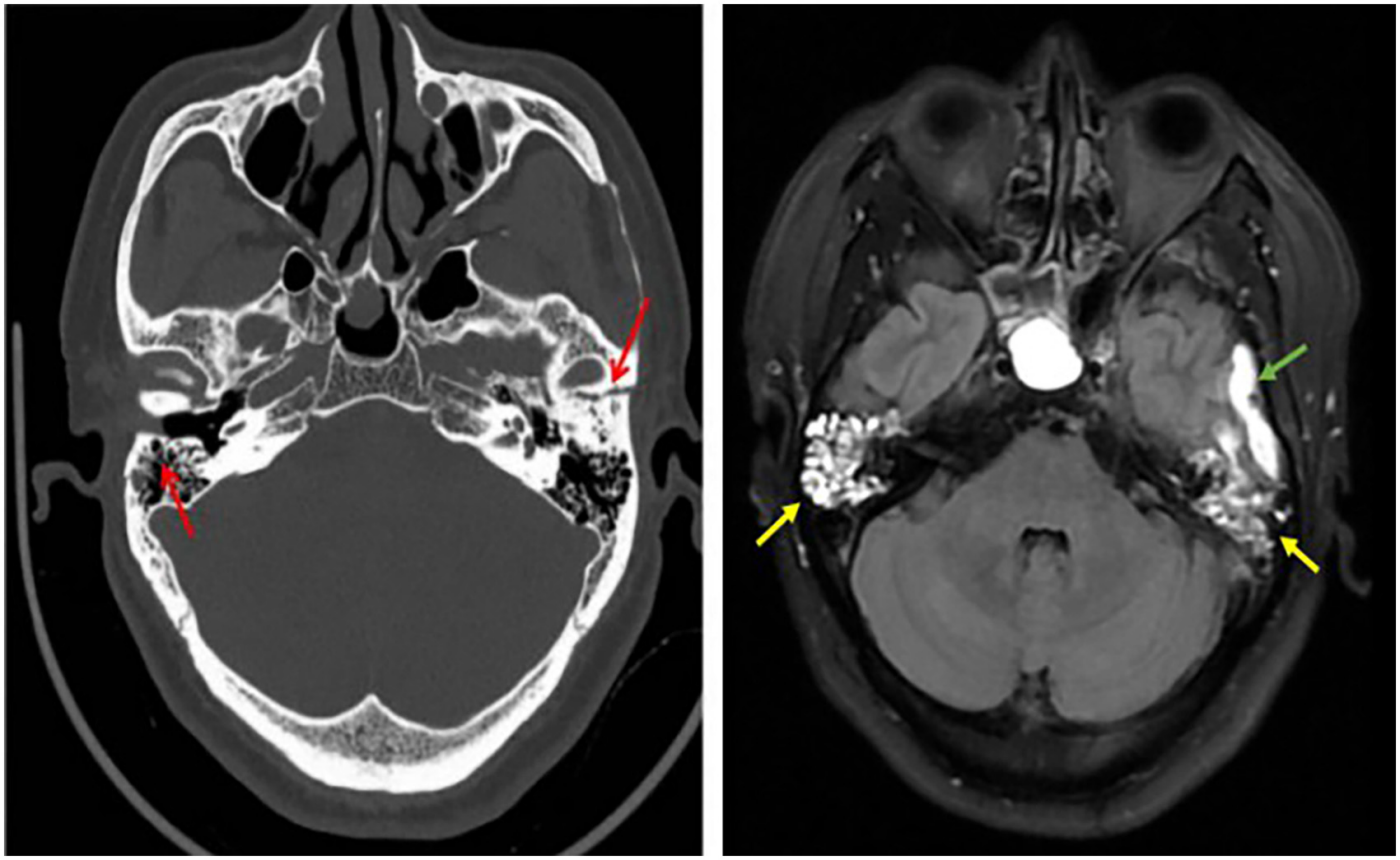
CT scan shows bilateral temporal bone fractures (indicated by the red arrow) and MRI reveals a left temporal lobe hematoma (indicated by the green arrow), fluid accumulation in bilateral mastoid processes and left sphenoid sinus (indicated by the yellow arrow).

A 47-year-old male was referred to our hospital 7 days post-traffic accident. He had experienced transient loss of consciousness with retrograde amnesia at the time of injury. He presented with bilateral facial nerve palsy lasting for 5 days, conductive hearing loss (air-bone gap of 15 dBHL on pure-tone audiometry), hypogeusia, and bilateral lower extremity hypoesthesia. The patient received dexamethasone intravenously (10 mg daily) for 4 consecutive days at the initial hospital, but no improvement in facial palsy was observed. He had undergone herniorrhaphy 30 years prior. He had no other significant medical history, notably no hypertension, diabetes, or other chronic conditions. He reported no smoking or alcohol use. Physical examination revealed absence of forehead wrinkles, incomplete bilateral eye closure and flattened bilateral nasolabial folds; the patient was unable to show teeth (Figure 1), corresponding to House-Brackmann grade V. Muscle strength is 5/5 in all four limbs. MRI revealed a left temporal lobe hematoma, fluid accumulation in bilateral mastoid processes and left sphenoid sinus. The CT scan demonstrated bilateral temporal bone fractures, but without involvement of the facial nerve canal (Figure 2). Facial nerve electroneuronography (ENoG) showed decreased CMAP amplitudes in the ocular and oral branches of the bilateral facial nerves (Table 1). Initially, temporal bone fracture was still considered to be the direct cause of facial palsy, and glucocorticoid therapy (dexamethasone 5 mg daily for 4 days) was continued while facial nerve decompression was planned as a consideration. However, a turning point occurred on day 5 after admission, when a neurologist noted diminished tendon reflexes (patellar) and strongly suspected Guillain-Barré syndrome (GBS). Subsequently, a cerebrospinal fluid (CSF) test and anti-ganglioside antibody test were performed. CSF analysis showed protein-cytological dissociation (protein: 71.1 mg/dL; cell count: 27/μL), anti-ganglioside antibody test revealed positive anti-GM2 IgM. Based on the medical history and laboratory findings, the patient was diagnosed with BFP, a rare variant of GBS.

**TABLE 1:** Electroneuronography (ENoG) amplitude values.

CMAP amplitudes(mV)	Ocular branches	oral branches
Left	0.12	0.1
Right	0.15	0.1

Plasma exchange was administered. A total of 4 sessions were performed at 2- to 3-day intervals. Fresh frozen plasma was used as the replacement fluid, with replacement volumes of 2300 mL, 2000 mL, 2800 mL, and 2760 mL, respectively, calculated from the patient’s body weight and hematocrit. Clinical improvement of facial palsy was first noted after the third session. During the fourth session, the patient developed acute adverse reactions, including profuse diaphoresis, nausea, fecal and urinary incontinence, and circumoral paresthesia. The procedure was immediately terminated. Vital signs at the time of onset were as follows: blood pressure 120/50 mmHg, temperature 36.7 °C, and heart rate 84 bpm. Serum calcium was normal (2.24 mmol/L), and no skin rash was observed. Neurological examination revealed no pathological reflexes or meningeal signs. The patient’s symptoms gradually resolved following intravenous infusion of Ringer’s lactate solution and slow intravenous push of calcium gluconate.

**FIGURE 3:**
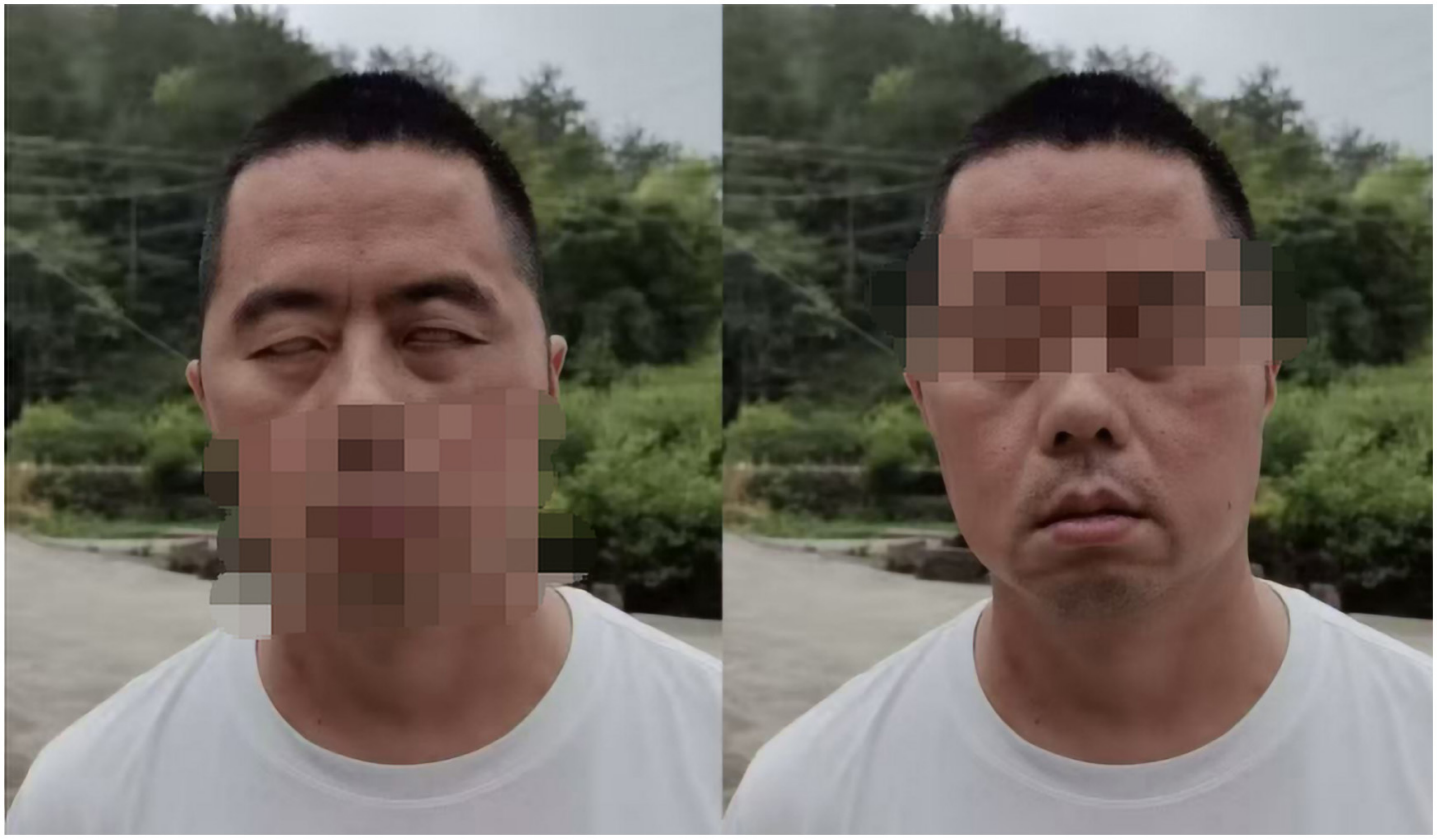
Two months post-injury, the patient presented improvement in the nasolabial fold flattening, but still incomplete eye closure.

**FIGURE 4:**
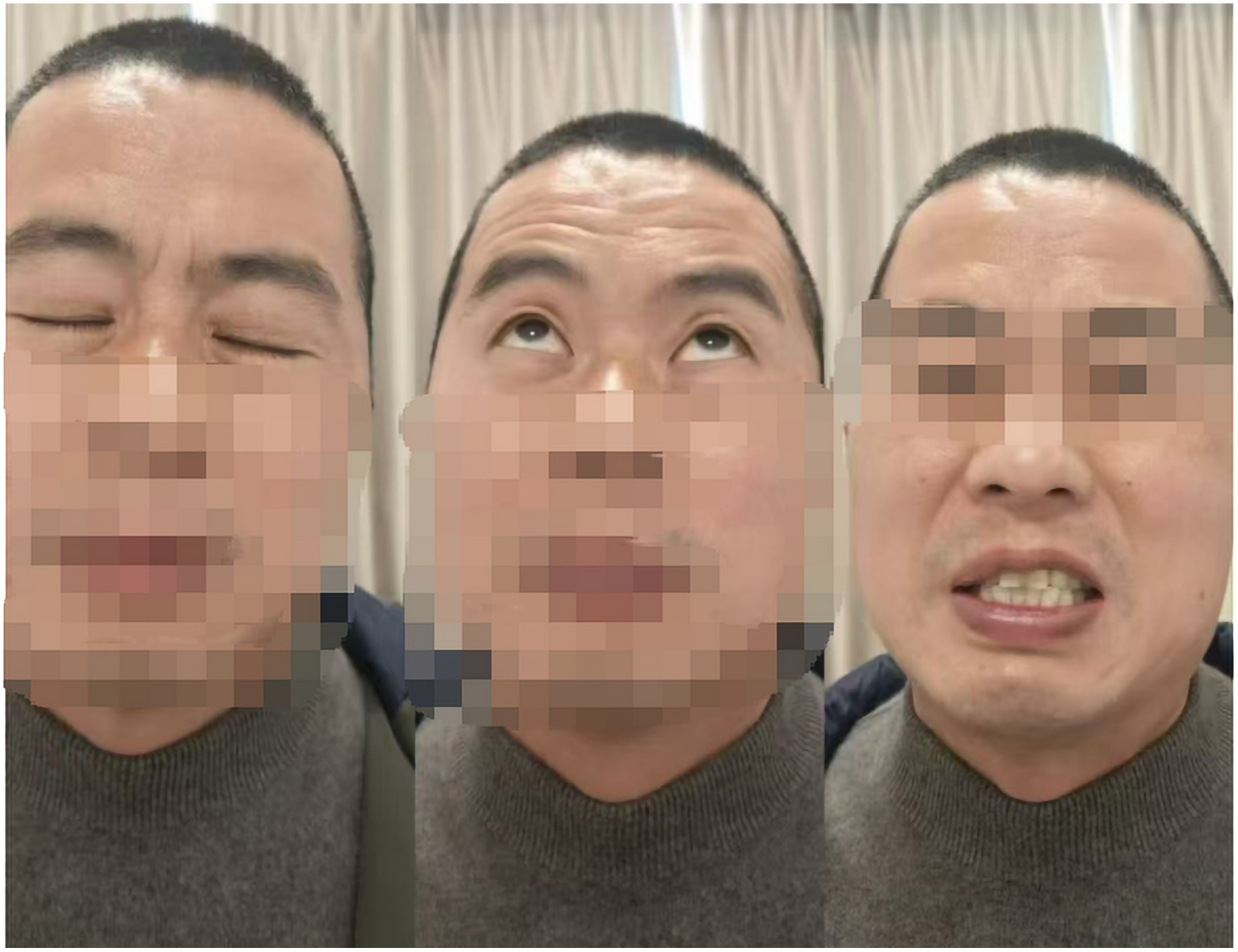
Six months post-injury, the patient showed complete eye closure, normal forehead wrinkles and nasolabial folds.

Two months post-trauma, oral competence during drinking was essentially restored; however, complete eye closure and forehead wrinkling remained suboptimal (Figure 3). 6 months post-injury, the patient achieved complete eye closure without scleral show, symmetric tooth display, and normal forehead wrinkles, corresponding to House-Brackmann grade I (Figure 4). The patient was satisfied with the functional recovery achieved. A timeline of the clinical course is presented in Supplementary Figure 1.

## 3 Discussion

### 3.1 Trauma and bilateral facial paralysis

Bilateral facial paralysis following trauma is rare and can be categorized into immediate and delayed onset. Immediate facial paralysis often indicates severe injury to facial nerve, like entrapment, laceration, contusion, crushing, or traction of the facial nerve. Delayed facial nerve paralysis typically occurs 2–21 days after the trauma and is more common than immediate onset, associated with edema, arterial or venous thromboses, or bleeding into the facial nerve bony canal that leads to external compression of the nerve (Undabeitia et al., 2013). Bilateral facial paralysis after trauma is a rare entity, with the literature confined to isolated case reports. Abrahão et al. (2021) performed a facial nerve decompression surgery on a patient with bilateral facial paralysis following trauma, with partial recovery of facial paralysis during follow-up. Interestingly, the temporal bone fracture line in this reported case closely resembled that of our patient. Although surgery was not ultimately carried out, the experience reported in this literature strengthened our consideration of it as a viable therapeutic option for this case. Medha et al. (2020) treated a patient with bilateral facial paralysis after trauma with corticosteroid and physiotherapy, showing satisfactory recovery after six months of follow-up. These reports indicate that the injury is the direct cause of the facial paralysis. Although our patient also presented with hypogeusia and conductive hearing loss, both of these symptoms could be adequately explained by local injury. Consequently, we remained confident in the accuracy of our diagnosis and did not question its validity.

### 3.2 Trauma and GBS

Guillain-Barré syndrome is typically triggered by infections, while post-traumatic GBS has likewise been documented solely in isolated case reports (Chen et al., 2021; Grote et al., 2021; Hendawi and Zavatsky, 2015). These reported cases of post-traumatic GBS usually show an onset window of 1–2 weeks. The physiopathology of GBS after trauma is still unclear. It may be related to immune stimulation caused by trauma, resulting in an aberrant autoimmune response to peripheral nerves and their spinal roots (Hughes and Cornblath, 2005). Grote et al. (2021) encourage clinicians to register any cases of post-traumatic or post-surgical GBS with international registers to raise awareness. Although the patient denied any history of respiratory or gastrointestinal tract infections, no etiological workup was performed. therefore, subclinical infection cannot be definitively excluded. Furthermore, the temporal interval between the onset of facial palsy and the preceding trauma was shorter than any previously reported immune-mediated post-traumatic GBS case. Consequently, a definitive etiological attribution of the patient’s disease could not be established.

### 3.3 Differential diagnosis of bilateral facial palsy

Bilateral facial nerve palsy is uncommon, occurring in 0.3%–2% of all facial palsies (D’Amore et al., 2012). Keane (1994) analyzed the causes in 43 patients with bilateral facial paralysis and found that the most common condition is Bell’s palsy (10 cases), followed by tumors (9 cases), and then GBS and infections (including tuberculosis, syphilis, and HIV). Neurosarcoidosis and neuroborreliosis should also be considered in the differential diagnosis. This patient had no involvement of other organs such as the lungs, eyes, or lymph nodes, and the disease course was acute and monophasic, thus ruling out sarcoidosis. There was no history of rash or tick bite prior to onset, and the absence of lymphocytosis in the cerebrospinal fluid examination excludes Lyme disease. Imaging examinations (Chest and abdominal CT scan, head MRI scan), and blood tests ruled out tumors and infectious diseases.

### 3.4 BFP, bifacial weakness with paresthesias

Guillain-Barré syndrome has diverse subtypes, and BFP is a rare variant characterized by rapidly progressive bilateral facial weakness in the absence of other cranial nerve involvement, ataxia, or limb weakness, many patients also experience distal limb paresthesia (Wakerley and Yuki, 2015). In Charous and Saxe (1962) reported a case of isolated facial paralysis with CSF protein-cytological dissociation, without involvement of other cranial nerves or limbs. Symptoms resolved after 3 weeks. They were the first to associate bilateral facial paralysis with GBS. Thirty years later, Ropper described four subtypes of GBS, including BFP (Ropper, 1994). In 2009, to determine the clinical features of BFP, Susuki et al. (2009) reviewed over 8,600 serological samples and medical histories of neurological diseases from their neuroimmunology laboratory, ultimately diagnosing 22 patients with BFP. All patients presented with CSF protein-cytological dissociation. Among them, 86% initially experienced limb numbness, indicating that subtle symptoms of generalized polyneuropathy (paresthesia) are a key feature of BFP. Mild distal limb paresthesia is a symptom easily overlooked by both clinicians and patients. Our patient’s sensory disturbances were not fully appreciated during the initial evaluation until detecting diminished tendon reflexes. In fact, limb paresthesia along with diminished or absent tendon reflexes is sufficient to distinguish BFP from most other causes of facial paralysis. Among the 22 patients, 82% had a history of preceding infections, indicating that infection remains the primary trigger, consistent with classic GBS. Our patient, with no history of preceding infection, is the first reported case of post-traumatic BFP meeting the strict diagnostic criteria proposed by Wakerley and Yuki. Eleven patients experienced dysgeusia. While classic GBS rarely involves taste abnormalities, dysgeusia is relatively common in Bell’s palsy and facial paralysis caused by trauma. Five patients tested positive for anti-GM2 IgM antibodies, with no anti-ganglioside IgG antibodies detected. Our patient was positive for anti-GM2 IgM.

### 3.5 Therapy

Intravenous Immunoglobulin Therapy (IVIG) and plasma exchange are standard treatments for GBS. In a study by Susuki et al. (2009), six patients received IVIG, four underwent plasma exchange, and three received steroid therapy. Except for one patient who still had severe facial weakness at the 10th month, most patients had a favorable prognosis. Our patient underwent plasma exchange and showed good recovery. According to previous literature, it is difficult to conclude that IVIG and plasma exchange are superior to observation. Although BFP typically has a favorable prognosis, patients with classic GBS may initially present with neurological symptoms similar to BFP and later progress to quadriplegia (Keane, 1994). Therefore, close monitoring symptoms of patients presenting with BFP is necessary, the standard treatments for GBS are reasonable. Plasma exchange was more cost-effective than immunoglobulin therapy, and the patient opted for this treatment.

Common adverse reactions associated with plasmapheresis include hypocalcemia-related reactions, anaphylaxis, and vasovagal responses. During the fourth session, the patient developed an adverse reaction that resolved largely after discontinuation of the procedure and administration of intravenous fluids and calcium. The absence of significant hypotension, tachycardia, or skin rash made anaphylaxis unlikely, and the lack of bradycardia further argued against a vasovagal etiology. Although the serum total calcium level was within normal limits, ionized calcium was not measured at the time of the event. Notably, the circumoral paresthesia, a hallmark of hypocalcemia, resolved following calcium administration, which strongly favors hypocalcemia as the underlying cause.

## 4 Limitations of this case report

Although diagnostic delayed several days inherent to the initial anchoring bias, fortunately, the patient achieved a relatively good outcome. Several limitations should be acknowledged.

A key limitation is the absence of four-limb motor and sensory nerve conduction studies, as well as electromyography data. The 2-day interval from trauma to facial palsy onset in our case is shorter than that of any previously reported immune-mediated post-traumatic GBS case. Yet this 2-day interval coincides with the timing of structural facial neuropathy from temporal bone fracture. This temporal discordance suggests two overlapping processes rather than a single autoimmune trigger: early structural injury (day 2) and a later demyelinating GBS process, indicated by diminished reflexes at day 12 within the expected immune latency window. Critically, facial ENoG alone cannot distinguish these entities. Complete limb electrophysiological studies are essential to determine whether the facial palsy reflects structural damage alone or a superimposed demyelinating process.

Another significant limitation is the absence of confirmatory microbiological or serological testing for potential infectious triggers. Anti-GM2 IgM seropositivity is a specific and well-documented marker of prior CMV infection and not merely a nonspecific autoimmune finding. Anti-GM2 IgM is significantly more prevalent in GBS patients with prior CMV infection than in those without (Jacobs et al., 1997). Regrettably, we did not perform CMV-specific IgM/IgG serology or viral PCR, and we excluded an infectious etiology based solely on the patient’s reported history of absent respiratory or gastrointestinal symptoms, and the absence of recent vaccinations. The possibility remains that an undetected CMV infection could have triggered the immune response.

## 5 Conclusion

Post-traumatic GBS has been documented in previous studies, but only in the context of classic ascending GBS with generalized limb weakness. This is the first case of post-traumatic BFP meeting the strict diagnostic criteria proposed by Wakerley and Yuki. We emphasize that in patients with post-traumatic bilateral facial nerve paralysis, a comprehensive neurological examination is essential for accurate diagnosis and to avoid unnecessary medications or surgery.

## Data Availability

The original contributions presented in the study are included in the article/Supplementary material, further inquiries can be directed to the corresponding author.

## Supplementary material

Supplementary Figure 1 The timeline of the clinical course.

## References

[B1] Abrahão N. M. Guimarães G. C. Castilho A. M. da Silva V. A. R. (2021). Bilateral facial paralysis secondary to temporal bone trauma: A case report and literature review. *Clin. Case Rep.* 9:e04272. 10.1002/ccr3.4272 34188929 PMC8218326

[B2] Charous D. I. Saxe B. I. (1962). The Landry-Guillain-Barre syndrome. Report of an unusual case, with a comment on Bell’s palsy. *N. Engl. J. Med.* 267 1334–1338. 10.1056/NEJM196212272672602 14020273

[B3] Chen J. Ma J. X. Zuo C. H. Zhang Q. Chen H. T. Ma X. L. (2021). Severe Guillain-Barré syndrome after surgery for multiple fractures: A rare case report with a 5-year follow-up and a brief review of the literature. *BMC Musculoskelet. Disord.* 22:8. 10.1186/s12891-020-03864-4 33397348 PMC7781168

[B4] D’Amore A. Viglianesi A. Cavallaro T. Chiaramonte R. Muscoso E. G. Giuffrida S. et al. (2012). Guillain-Barré syndrome associated with acute onset bilateral facial nerve palsies. A case report and literature review. *Neuroradiol. J.* 25 665–670. 10.1177/197140091202500604 24029178

[B5] Grote H. Sim N. K. Rinaldi S. Carswell C. (2021). Post-traumatic (and postsurgical) Guillain-Barré Syndrome: A rare, but treatable entity. *BMJ Case Rep.* 14:e238116. 10.1136/bcr-2020-238116 33558381 PMC7872914

[B6] Hendawi T. Zavatsky J. M. (2015). Guillain-Barré syndrome after pelvic fracture fixation: A rare cause of postoperative paralysis. *Spine* 40 E372–E374. 10.1097/BRS.0000000000000779 25774469

[B7] Hughes R. A. Cornblath D. R. (2005). Guillain-Barré syndrome. *Lancet* 366 1653–1666. 16271648 10.1016/S0140-6736(05)67665-9

[B8] Jacobs B. C. van Doorn P. A. Groeneveld J. H. Tio-Gillen A. P. van der Meché F. G. (1997). Cytomegalovirus infections and anti-GM2 antibodies in Guillain-Barré syndrome. *J. Neurol. Neurosurg. Psychiatry* 62 641–643. 10.1136/jnnp.62.6.641 9219756 PMC1074154

[B9] Keane J. R. (1994). Bilateral seventh nerve palsy: Analysis of 43 cases and review of the literature. *Neurology* 44 1198–1202. 10.1212/wnl.44.7.1198 8035915

[B10] Medha K. K. Gupta M. Gupta M. (2020). Post-traumatic bilateral longitudinal temporal bone fracture with bilateral facial nerve palsy: A rare case. *BMJ Case Rep.* 13:e233728. 10.1136/bcr-2019-233728 32086330 PMC7046368

[B11] Ropper A. H. (1994). Further regional variants of acute immune polyneuropathy. Bifacial weakness or sixth nerve paresis with paresthesias, lumbar polyradiculopathy, and ataxia with pharyngeal-cervical-brachial weakness. *Arch. Neurol.* 51 671–675. 10.1001/archneur.1994.00540190051014 8018039

[B12] Susuki K. Koga M. Hirata K. Isogai E. Yuki N. A. (2009). Guillain-Barré syndrome variant with prominent facial diplegia. *J. Neurol.* 256 1899–1905. 10.1007/s00415-009-5254-8 19633904

[B13] Undabeitia J. Liu B. Pendleton C. Nogues P. Noboa R. Undabeitia J. I. (2013). Bilateral traumatic facial paralysis. Case report. *Neurocirugia* 24 225–228. 10.1016/j.neucir.2013.01.003 23541180

[B14] Wakerley B. R. Yuki N. (2015). Isolated facial diplegia in Guillain-Barré syndrome: Bifacial weakness with paresthesias. *Muscle Nerve* 52 927–932. 26315943 10.1002/mus.24887

